# A Cardiology Handbook App to Improve Medical Education for Internal Medicine Residents: Development and Usability Study

**DOI:** 10.2196/14983

**Published:** 2020-04-16

**Authors:** Asad Torabi, Abhishek Khemka, Pantila V Bateman

**Affiliations:** 1 Indiana University School of Medicine Indianapolis, IN United States; 2 Krannert Institute of Cardiology Indiana University School of Medicine Indianapolis, IN United States

**Keywords:** mobile learning, medical reference app, cardiology, internal medicine

## Abstract

**Background:**

At most institutions, internal medicine residents struggle with balancing clinical duties and learning opportunities, particularly during busy cardiology ward rotations. To improve learning experiences for residents, we helped develop a cardiology handbook app to supplement cardiology education.

**Objective:**

The aim of this study was to report the development, implementation, and preliminary impact of the Krannert Cardiology Handbook app on graduate medical education.

**Methods:**

In June 2017, 122 residents at Indiana University were invited to download the digital handbook in the Krannert app. The Krannert app featured a total of 13 chapters written by cardiology fellows and faculty at Indiana University. Residents were surveyed on their self-reported improvement in cardiology knowledge and level of satisfaction after using the Krannert app. Residents were also surveyed regarding their preference for a digital handbook app versus a paper handbook.

**Results:**

Of the 122 residents, 38 trainees (31.1%) participated in survey evaluations. Among all respondents, 31 app users (82%) reported that the app helped improve their cardiology knowledge base. The app had an overall favorable response.

**Conclusions:**

The Krannert app shows promise in augmenting clinical education in cardiology with mobile learning. Future work includes adding new topics, updating the content, and comparing the app to other learning modalities.

## Introduction

Clinical education is a complex process. The need to strengthen the quality of medical education has led to educational innovation and novel instructional strategies such as simulation and mobile technology [1,2]. Medical reference apps are being recognized as an increasingly important asset to improve medical education. In a survey polling over 3000 residents, fellows, and staff in Accreditation Council for Graduate Medical Education (ACGME) programs, the most popular app types requested were those on textbook and reference material, in-training exam material, classification and treatment algorithms, and general medical knowledge [3]. Respondents in the survey also indicated a demand for higher-quality apps.

Despite the growing popularity of medical reference apps, there is insufficient data on the use and effectiveness of smartphones and mobile learning in graduate and undergraduate medical education [4-9]. Currently, most reference apps cover general medical knowledge, with some serving as a calculator. In cardiology, there are limited specialty-specific reference apps available [10].

A recent review demonstrates that published medical education research in cardiology is lacking [11]. In general, there is a paucity of resident-level teaching tools available in cardiovascular education. There are also limited publications covering narrow cardiovascular topics, including the evaluation of chest pain and myopericarditis [12,13], but no comprehensive teaching tools to cover common cardiac diseases. An easily accessible teaching tool in cardiology would be beneficial to residents and supplement their existing cardiology curriculum.

At our institution, residents (of internal medicine, medicine-pediatrics, and preliminary medicine) were polled about their learning experiences during their inpatient cardiology rotation. A review of their free-text responses indicated the need for further learning opportunities. After reviewing the needs assessment, our overarching goal was to modernize teaching in cardiology and improve bedside learning by creating a digital handbook as a practical reference for trainees. Learning objectives were accomplished by residents going through the digital handbook individually at their own pace during the rotation. The aim was to provide trainees with an easily accessible and reliable source to better understand cardiac disease pathology, diagnostics, and management. This intervention was not designed to replace didactics or traditional bedside learning but rather to supplement them. In this paper, we report the development, implementation, and preliminary evaluation of the Krannert Cardiology Handbook app.

## Methods

### Overview

The Krannert app was piloted in 2017 as a cardiology reference tool for residents beginning their inpatient cardiology months at Indiana University, an urban academic medical center. A total of 122 residents (categorical, medicine-pediatrics, and preliminary) from 2017-2018 were invited to download the app before and during their orientation for an inpatient cardiology rotation. The study was deemed exempt from Institutional Review Board (IRB) review by the Kuali Coeus IRB Office of Research Compliance from Indiana University. Learners were provided with informed consent and no personal information was collected.

### Needs Assessment

For curriculum development, we conducted a targeted needs assessment at our own institution. We reviewed free-text responses from an electronic survey, via MedHub, asking residents to describe their learning experiences (ie, lectures, conferences, case discussions, overall quality of faculty teaching, bedside teaching, adequate time for reading and studying) during their clinical rotation. The results of this assessment highlighted difficulties in balancing service and educational duties on a clinically demanding service as seen in other specialties [14]. The residents noted a lack of adequate learning opportunities and specifically requested more teaching in electrocardiographic interpretation and echocardiography. In response to these comments, we created the Krannert app curriculum to help improve educational experience.

### Curricular Design

We arranged the digital handbook into 13 chapters, which were written by cardiology faculty members and cardiology fellows from the Krannert Institute of Cardiology at Indiana University. All materials were peer-reviewed by faculty members (ie, senior attendings) who work with medical trainees, including fellows, residents, and medical students. All attendings are board certified in cardiovascular disease and, depending on their specific subspecialty, they may also be board certified in internal medicine, interventional cardiology, electrophysiology, echocardiography, and nuclear cardiology. The cardiology fellows were board certified in internal medicine and in the process of completing their 3-year training in general cardiovascular disease in an ACGME accredited program at Indiana University. The names of the authors were listed in each chapter.

**Figure 1:**
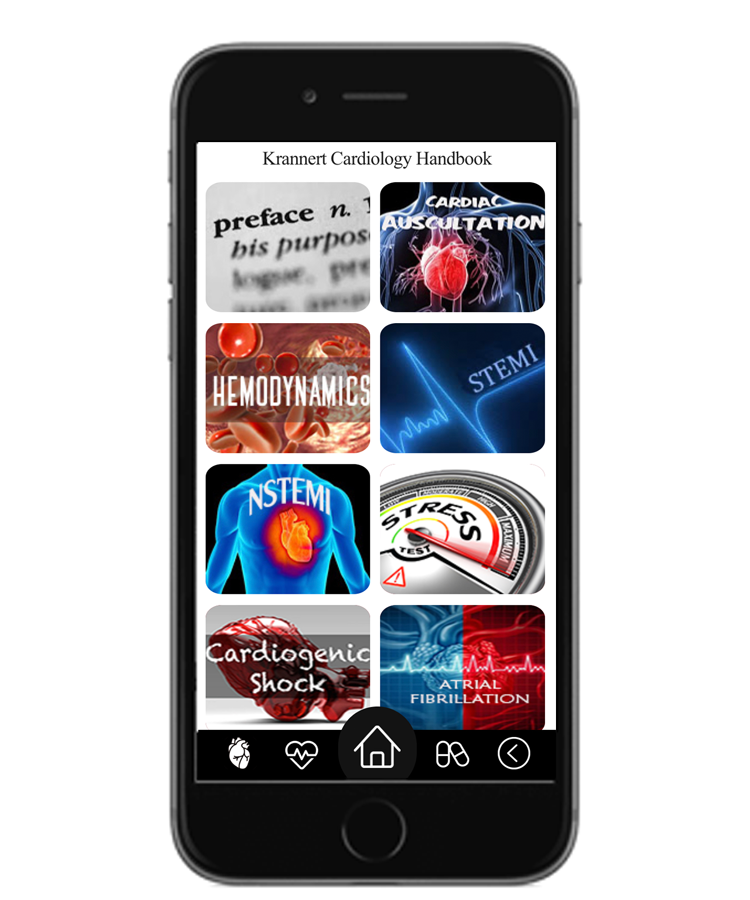
Table of contents of the Krannert Cardiology Handbook app. Chapters not visualized in the figure are tachyarrhythmia, bradyarrhythmia, syncope, pulmonary hypertension, cardiogenic pharmacology, and introduction to echocardiography.

In the beginning, the reading content and specific topics were selected by the authors, who also serve as key clinical educators within the Cardiology Division. Specific topics were chosen based on common diagnoses for patients admitted to the cardiology care unit as well as for patients from the cardiology consultation services (Figure 1). It was also based on findings from our targeted needs assessment. The app content was written in a succinct, outline format to maintain brevity and serve as a quick reference along with basic classification and treatment algorithms. The app as a resource was designed to provide practical knowledge and not as a reference for in-training exam material. We made a point to include a guide on hemodynamics to review the fundamentals of right heart catheterization, valvular and pericardial pathology, and intra-aortic balloon pump because these are common topics that general medicine residents may not commonly encounter on other hospital rotations. Learning material involving ST-elevation myocardial infarction, non–ST-elevation myocardial infarction, cardiogenic shock, and arrhythmias, with potential complications and management, were included to help trainees feel more comfortable managing their acutely ill patients. Content developed by fellows was written from a peer teaching perspective, and many chapters included figures, tables, and images as well as references for deeper understanding of basic concepts (see Table 1 for app objectives and Multimedia Appendix 1 for sample content). After the content was written, app development by The Center for Physician Education at Indiana University Health took approximately 100 hours. The cost was US $10,000.

**Table 1:** Krannert Cardiology Handbook app objectives.

Objective	Description
Acute coronary syndrome	List features of unstable angina/non–ST elevation myocardial infarction from ST elevation myocardial infarction List indications and complications of heart catheterization and intra-aortic balloon pump placement. Recognize appropriate pressure wave forms Identify complications postmyocardial infarction List common antianginal medications. List indications and contraindications for thrombolytics and anticoagulants List indications for ionotropic stimulation for stress testing
Miscellaneous	Identify workup for syncope List types of pulmonary hypertension
Cardiac arrhythmias	Identify electrocardiographic abnormalities and manage supraventricular and ventricular arrhythmias Recognize and manage bradyarrhythmias
Cardiac failure	Recognize and manage patients with acute decompensated heart failure including acute pulmonary edema and hypotension; understand use of inotropic and afterload reducing agents Differentiate cardiogenic shock from other types of shock
Hemodynamics	Recognize and manage valvular heart disease, including emphasis of physical exam findings indicative of severe valvular disease. Recognize need for surgical correction Identify basic views of 2D echocardiogram and examples of valvular regurgitation and stenosis Identify types of pericardial disease

The handbook was released as an app on Apple’s App Store [15] in June 2017 and subsequently on Google Play [16] in September 2017; both are available free of charge. The mobile app was introduced to all medicine residents at Indiana University as a single iteration, by an email sent from the internal medicine department. The app could be utilized by trainees on any rotation but was targeted toward residents on the cardiology service. One week prior to starting the cardiology service, residents received an email with orientation materials, which included information regarding the app, advertised as a reference tool.

### Survey Design

To evaluate the Krannert app, we gathered data throughout the 2017-2018 academic year with an anonymous self-generated survey. This was an open survey built into the app, completely voluntary, and advertised by the internal medicine department through email. No incentives were offered to fill in the survey. The questions were not randomized, and the target population was residents who used the app. The questionnaire had not been tested before, as this was a pilot study. Respondents were able to review and change answers before submission. We only analyzed completed surveys, and we did not weigh survey items or use propensity scores. We did not use IP (Internet Protocol) checks to evaluate whether responses were unique because learners were allowed to use the app on any device that they wished to use, and we did not have respondents enter any personal information.

### Outcome Measures

We were interested in three outcomes: (1) self-reported improvement in cardiology knowledge; (2) resident satisfaction with the handbook; and (3) resident learning preference (ie, the Krannert app versus a traditional paper handbook). Responses were recorded using a 5-point Likert-type scale from 1=“strongly disagree” to 5=“strongly agree” (Multimedia Appendix 2). We used descriptive statistics to summarize all data in survey item responses. Responses like “agree” or “strongly agree” to a statement were considered positive. We used self-reported learning results to measure the effectiveness of the app in achieving the stated educational objectives.

## Results

### Participants

Of the 122 residents invited to download the app, 38 (31% completion rate) participated in the survey.

### Medical Trainee–Perceived Learning

We used a single survey item from 38 respondents to assess self-reported learning after using the app. The majority of residents (n=31; 82%) reported that they agreed or strongly agreed that the app helped them improve their cardiology knowledge base (Multimedia Appendix 3, item 1).

### Medical Trainee Satisfaction

Among all respondents, 90% (n=34) indicated the app was easy to use and 87% (n=33) reported that the app content was delivered in a user-friendly manner. Overall, trainees found the app to be acceptable (n=33; 87%). From all respondents, 76% (n=29) reported that the amount of app content was appropriate and 71% (n=27) reported that the app met their educational needs in cardiology (Multimedia Appendix 3, items 2-6).

### Learning Preference

Of the 38 respondents, 74% of medical trainees (n=28) reported a preference for the mobile app over a traditional paper cardiology handbook. Nearly 24% (n=9) were neutral in their response to this survey item (Multimedia Appendix 3, item 7).

## Discussion

### Principal Findings

We introduced a novel educational app to improve learning opportunities for our trainees on their cardiology rotation and received favorable results. Easy access to the app as a teaching tool appeared to play a key role in improving their learning experience at our institution. Although 74% (28/38) of trainees preferred learning through mobile resources over traditional paper resources, other trainees did not have a strong inclination. It is our opinion that the benefits of a digital handbook include convenience and transportability to help answer clinical questions at the point of need at a cost that is fairly lower than printing a textbook. Learning resources should be readily accessible, and use of a mobile app provides trainees with this benefit. Mobile devices can also be used to customize educational materials. The Krannert app curriculum can be used by a variety of learners, including students in undergraduate medical education, and can serve as a teaching aid for institutions teaching adult cardiology. An educational handbook app may also be beneficial to other medical specialties, in general.

Our relatively positive experiences with a mobile app are comparable to other studies that introduced mobile textbook apps to trainees [17-19]. Hardyman et al [17] complied medical textbooks into an app called iDoc. Using self-reported patient encounters, the house staff in Wales reported an improvement in their efficiency, effectiveness, and timeliness in patient care. Therefore, we believe that the use of medical apps in education will likely continue to be helpful and popular among medical trainees. However, the need is great for high-quality medical apps that are accurate and up-to-date. There is also a growing need to learn more about how medical apps can impact and improve medical education. This is a distinctively unique learning platform in the ever-expanding digital world.

### Strengths and Limitations

We had some limitations in our study. Because this is a pilot feasibility study, the sample size is small. We did not design the study to compare results between a control group and an intervention group. We did not assess measurable gains in learner’s knowledge with pre- and postknowledge assessments, which may yield different results than self-reported outcomes. Additionally, we did not compare the results of the app to other learning experiences, such as bedside teaching, lectures, or case conferences. Tracking the number of app downloads by our own residents was limited due to privacy restrictions from Apple and Google. We also did not assess whether there were certain topics that learners felt were ineffective or had difficulties with using the app interface.

### Recommendations and Future Research

Further research should study larger populations of residents across multiple institutions and include written practical knowledge assessments. Future work will also include a comparison of the app with other learning modalities or experiences and assess whether learning from the app leads to sustained learning. Future steps in improving our app include adding new topics such as preventive cardiology, congenital heart disease, and cardio-oncology, updating the content, and making the app more interactive. We plan to review the app content annually and use the survey link within the app to directly incorporate resident feedback into the app content and layout design.

### Conclusions

The development of a digital handbook app improved medical education for trainees in cardiology and appeared to play a key role in improving their learning experience at our institution. Future steps in improving our app include adding new topics, updating the content, and making the app more interactive. Trainees from other programs may also benefit from having an educational handbook app in cardiology as well as other medical specialties.

## Appendix

Multimedia Appendix 1 Screenshots of the Krannert Cardiology Handbook app.

Multimedia Appendix 2 A postcurriculum survey regarding thoughts on the Krannert app and its cardiology curriculum.

Multimedia Appendix 3 Survey results for self-reported learning (item 1), trainee satisfaction (items 2-6), and learning preference (item 7) (N=38).

## Abbreviations

ACGME: Accreditation Council for Graduate Medical Education
IP: Internet Protocol
IRB: Institutional Review Board
