# Supplementary material for: A Cardiology Handbook App to Improve Medical Education for Internal Medicine Residents: Development and Usability Study
Source: JMIR Med Educ. 2020 Apr 16;6(1):e14983. doi: 10.2196/14983 (PMC7193443; doi:10.2196/14983)

Supplementary Content 1

Screen Shot of Krannert Cardiology Handbook App to demonstrate Stress Testing Chapter, 2019.


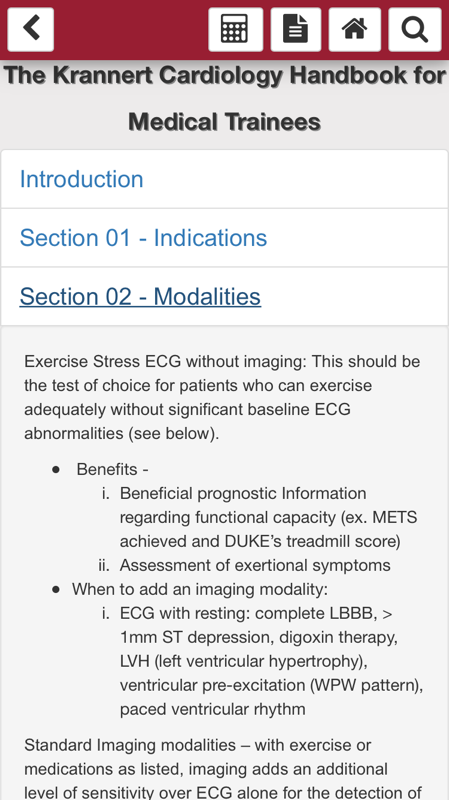


Supplementary Content 2

Screen Shot of Krannert Cardiology Handbook App to demonstrate Echocardiography and Wall Motion Chapter, 2019.


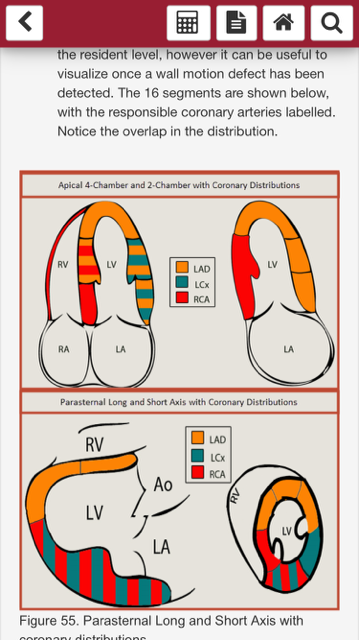

Supplement: Multimedia Appendix 1 [file mededu_v6i1e14983_app1.docx]
