# Supplementary material for: A Cardiology Handbook App to Improve Medical Education for Internal Medicine Residents: Development and Usability Study
Source: JMIR Med Educ. 2020 Apr 16;6(1):e14983. doi: 10.2196/14983 (PMC7193443; doi:10.2196/14983)
Supplement: Multimedia Appendix 2 [file mededu_v6i1e14983_app2.docx]

Krannert App Post Survey

This survey is a postcurriculum survey regarding thoughts on the Krannert app and its cardiology curriculum.

Questions

1. The amount of app content was appropriate.

| Strongly Agree | Agree | Neutral | Disagree | Strongly Disagree |
| --- | --- | --- | --- | --- |

1. The app helped me improve my cardiology knowledge base.

| Strongly Agree | Agree | Neutral | Disagree | Strongly Disagree |
| --- | --- | --- | --- | --- |

1. The app met my educational needs in cardiology.

| Strongly Agree | Agree | Neutral | Disagree | Strongly Disagree |
| --- | --- | --- | --- | --- |

1. The app content was delivered in a user-friendly manner

| Strongly Agree | Agree | Neutral | Disagree | Strongly Disagree |
| --- | --- | --- | --- | --- |

1. The app was easy to use.

| Strongly Agree | Agree | Neutral | Disagree | Strongly Disagree |
| --- | --- | --- | --- | --- |

1. I would prefer the Krannert mobile app over a traditional paper cardiology handbook.

| Strongly Agree | Agree | Neutral | Disagree | Strongly Disagree |
| --- | --- | --- | --- | --- |

1. Overall, I found the app to be acceptable.

| Strongly Agree | Agree | Neutral | Disagree | Strongly Disagree |
| --- | --- | --- | --- | --- |
