# Supplementary figures and images for: A Cardiology Handbook App to Improve Medical Education for Internal Medicine Residents: Development and Usability Study
Source: JMIR Med Educ. 2020 Apr 16;6(1):e14983. doi: 10.2196/14983 (PMC7193443; doi:10.2196/14983)

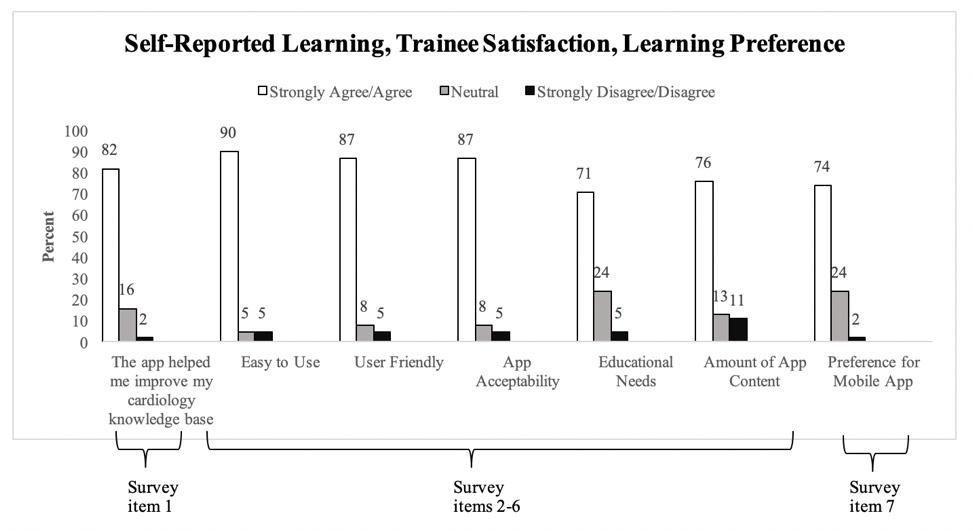

Supplement: Multimedia Appendix 3 [file mededu_v6i1e14983_app3.png]
